# The Association Between Lifestyle and Symptoms of Polycystic Ovary Syndrome: A Study Conducted on Taif University Medical Students

**DOI:** 10.7759/cureus.70169

**Published:** 2024-09-25

**Authors:** Shatha H Alziyadi, Wejdan A Alkhaldi, Rawan M Altowairqi, Lama A Alkhaldi, Thamer I Albaqami

**Affiliations:** 1 Department of Obstetrics and Gynecology, Taif University, Taif, SAU; 2 Medical School, Taif University, Taif, SAU

**Keywords:** insulin resistance, lifestyle behaviours, polycystic ovary syndrome (pcos), women’s health, medical students

## Abstract

Introduction

Polycystic ovary syndrome (PCOS) is a common endocrinological disorder affecting millions of women worldwide, often leading to significant physiological and psychological challenges among medical students because of unhealthy lifestyle behaviors.

Methods

A cross-sectional study was conducted among female medical students at Taif University in Saudi Arabia in January 2024 using an online questionnaire distributed via a Google form.

Results

Out of 243 participants, 23 students (9.5%) were diagnosed with PCOS. One hundred and fifty-six (64.9%) of females experienced skin symptoms, including acne and oily skin, mostly during exam periods. More than half (n=131, 54.4%) had generalized anxiety, and 42 students (17.4%) reported delays in the menstrual cycle. Significant associations were found between being diagnosed with PCOS and experiencing generalized anxiety (p=0.020), irregular menstrual cycles (p=0.004), having diabetes (p=0.006), family history of PCOS (p=0.001), and being aware of PCOS and lifestyle relation (p=0.022).

Conclusion

The study findings showed that Taif University medical students do not exhibit a noticeably increased prevalence of PCOS. Still, most students are at risk of developing PCOS due to the positive family history of PCOS and excessive generalized anxiety and females with irregularity in the menstrual cycle and those who have diabetes.

## Introduction

Polycystic ovary syndrome (PCOS) is a common endocrinological disease that affects nearly 116 million women worldwide. In 2021, the World Health Organization found women of reproductive age with PCOS experience compromised academic and professional success because of the effects of PCOS [1]. According to studies conducted in the United States, they found the prevalence of PCOS ranges from 5 to 7% [2]. The result of a survey among health sciences students in South India to know the proportion and determinants of PCOS showed that 8.1% of participants had a PCOS diagnosis. Of the remainder, 90.9% had a low risk of PCOS, and 9.1% had a high risk [3].

Moreover, in Oman, women with PCOS had a prevalence of 2.8% in hospital-based studies in 2010 [4]. Based on Rotterdam criteria to measure the prevalence of PCOS, they found that 14.1% of Iranian females have PCOS [5]. In Saudi Arabia, the prevalence of PCOS was revealed to be 32.5%, according to a study carried out among 719 women living in Madinah city [6]. Additionally, according to an online survey conducted at Princess Nourah bint Abdulrahman University in Saudi Arabia, 16% of women suffer from PCOS [7].

Furthermore, PCOS is a multifarious syndrome characterized by multiple features: menstrual irregularities, hyperandrogenism, and polycystic ovarian development [8]. There are several diagnostic criteria for PCOS; for example, in 2006, the Androgen Excess Society (AES) relied on the hyperandrogenism symptom as the main diagnosis for PCOS. Currently, the modified Rotterdam criteria is the most followed one, which diagnoses PCOS based on the existence of at least two of the following features: menstrual irregularity, polycystic ovarian morphology using ultrasound, hyperandrogenism (hirsutism, acne, and hair loss), and exclusion of any other clinical condition [9]. In addition to its effects on reproduction, PCOS is associated with several metabolic and psychiatric comorbidities that significantly impair the health of those who are affected [10].

Furthermore, medical students are faced with an extremely rigorous curriculum that could lead to stress with a sedentary lifestyle and altered dietary patterns might potentially raise the risk of developing PCOS. Nonetheless, studies focused on women with PCOS showed that medical school students with PCOS faced both the physiological and psychological effects of the disorder while studying [11-12].

Worldwide, PCOS significantly reduces women's quality of life [13]. It may be considered a risk factor for developing cancer [14]. Therefore, this study aimed to raise medical students' awareness of the relationship between their lifestyle and PCOS. In addition, will share recommendations based on the results to enhance health quality in Saudi Arabia.

## Materials and methods

Study design and settings

This is a cross-sectional study conducted from 15 January to 29 January 2024 at Taif University to investigate the prevalence of symptomatic PCOS among female medical students and its association with lifestyle behaviors.

Study population

Data was collected from 243 female medical students in the 1st to 6th year at Taif University from 15 to 29 January 2024 who were willing to participate. The sample size among 658 female medical students at Taif University was calculated using an online Raosoft sample size calculator (www.raosoft.com).

Data collection method and tool

Data was collected using an online questionnaire (Appendix 1) distributed through an online Google form. The questionnaire consisted of four main sections: consent to participate and included the academic year, diagnosis of PCOS, skin symptoms, and lifestyle characteristics, including tension, anxiety, menstrual cycle regularity, diabetes status, awareness of the relationship between lifestyle and PCOS, dietary habits, and exercise frequency. The online questionnaire uses a pilot test. The pilot study was conducted on six individuals to ascertain the tool's feasibility, applicability, and clarity. However, five people were excluded from the final analysis of the pilot study.

Ethical consideration

The survey was restricted to one response only using the participants' email, and personal information was kept undeclared to maintain accuracy and confidentiality. All participants signed a written form including all females studying in medical college at Taif University. The ethical approval was obtained from the Taif University Ethics Committee No. HAO-02-T-105 on 22 January 2024 (approval number 45-138).

Statistical analysis

Data collected was entered into an Excel sheet for data validation and was introduced to the Statistical Package of Social Sciences Software Program (SPSS), version 26.0 (IBM Corp., Armonk, NY) for statistical analysis. Categorical variables were expressed as counts and percentages. The Chi-square was used to assess the association between medical students' lifestyle characteristics (independent variables) and the prevalence of PCOS (dependent variable). A test was considered significant if the p-value was lower than 0.05.

## Results

**Figure 1:**
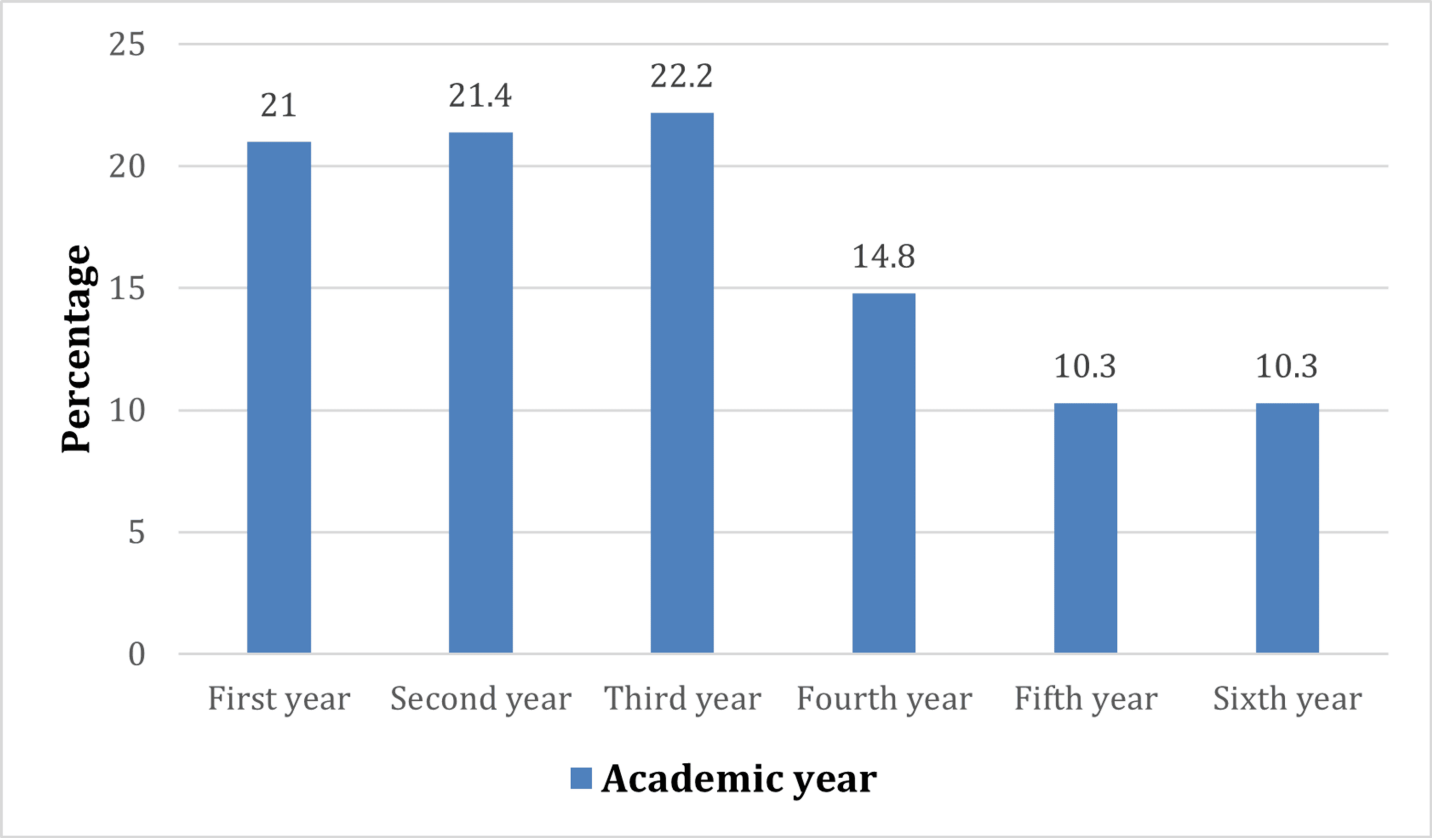
Academic year of included participants

The study included 243 female medical students, most of whom were from the first three years of university. The highest representation was in the third year, n=54 (22.2%), followed by the second year, n=52 (21.4%), then the first year, 51 (21%), as shown in Figure 1.

Table 1 shows the prevalence of PCOS and associated skin symptoms. Only 23 students (9.5%) were diagnosed with PCOS. Regarding skin symptoms, 156 students (64.9%) suffered from acne and oily skin, whereas 104 (43.0%) experienced an increase in skin symptoms during exam periods and 52 (21.5%) experienced them during the semesters. Additionally, 36 students (14.9%) experienced a blackish mark around their neck.

**Table 1:** Prevalence of PCOS and skin symptoms PCOS: Polycystic ovary syndrome.

Questions		N	Percentage
Have you been diagnosed with PCOS?	No	219	90.5
	Yes	23	9.5
Do you suffer from acne and oily skin recently?	No	86	35.5
	Yes	156	64.9
If yes, what time is it increased?	The previous answer is no	86	35.5
	During the exam period, only	104	43.0
	Along semester	52	21.5
Do you have a blackish mark around your neck?	No	206	85.1
	Yes	36	14.9
PCOS: polycystic ovarian syndrome, N: number			

Table 2 illustrates the lifestyle characteristics of medical students. About half of the participants, n=131, 54.4%, experienced excessive tension and anxiety during exams, and 76 (31.5%) had been dealing with these symptoms since the first year of study. About 42 (17.4%) reported a delay in their menstrual cycle, with 29 (12%) having a delay for one month and nine (3.7%) for two months. Only eight students (3.3%) have diabetes, 20 (8.3%) are obese, 41 (17%) are overweight, and 28 (11.6%) have a family history of PCOS. Furthermore, 113 students (46.9%) were aware of the relationship between lifestyle behavior and PCOS. The vast majority (n=203, 84.2%) reported a varied diet between healthy and unhealthy options.

**Table 2:** Medical students' lifestyle characteristics PCOS: Polycystic ovarian syndrome, BMI: Body mass index, N: Number.

Lifestyle characteristics		Count	%
Do you experience excessive tension and anxiety?	No	34	14.1
	Yes, during the exam, only	131	54.4
	Yes, since 1^st^ year, I have this problem	76	31.5
Is your menstrual cycle regular?	No	42	17.4
	Yes	200	82.6
If not, how long has it been delayed?	The last answer is yes	200	82.6
	One month	29	12.0
	Two months	9	3.7
	Three or more months	4	1.7
Do you have diabetes?	No	234	96.7
	Yes	8	3.3
If yes, HbA1c	The previous answer is no	234	96.7
	Normal	3	1.2
	Seven or above %	5	2.1
If yes, which type of diabetes?	The previous answer is no	234	96.7
	Type 1	5	2.1
	Type 2	3	1.2
	Gestational diabetes	0	0.0
Do you have PCOS in your family?	No	109	45.2
	Yes	28	11.6
	I do not know	104	43.2
BMI	Underweight (BMI<18.5 kg/m^2^)	32	13.3
	Normal (BMI 18.5–24.9 kg/m^2^)	148	61.4
	Overweight (BMI 25–29.9 kg/m^2^)	41	17.0
	Obese (BMI≥30 kg/m^2^)	20	8.3
How many times do you work out?	No, I did not	110	45.5
	Once	43	17.8
	Twice	40	16.5
	Three times	26	10.7
	More than three times	23	9.5
If the previous question is yes, what type of workout do you do?	The previous answer is no	110	45.5
	Walk	123	50.8
	Swimming	5	2.1
	Basketball	2	0.8
	Football	1	0.4
	Cycling	1	0.4
Have you heard about the relationship between lifestyle behavior and polycystic ovarian syndrome?	No	128	53.1
	Yes	113	46.9
What kind of food do you eat?	Healthy	10	4.1
	Vary between healthy and unhealthy	203	84.2
	Unhealthy	28	11.6

Table 3 shows crosstabulation between various lifestyle characteristics and the prevalence of PCOS. Significant associations were found between being diagnosed with PCOS and experiencing excessive tension and anxiety (p=0.020), irregular menstrual cycles (p=0.004), having diabetes (p=0.006), and a family history of PCOS (p=0.001) showed a higher prevalence of PCOS than who didn’t. Moreover, those experiencing PCOS had a higher association with the awareness of the relationship between lifestyle and PCOS than those who didn’t (p=0.022). Regarding dietary habits, participants reporting a healthy diet had a higher PCOS prevalence than those with varied or unhealthy diets (p=0.040).

**Table 3:** The association between medical students' life lifestyle characteristics and the prevalence of PCOS * Chi-square test; PCOS: Polycystic ovarian syndrome, BMI: Body mass index, N: Number, %: Percentage.

Questions		Diagnosed with PCOS				X^2^	P-value*
		No		Yes			
		N	%	N	%		
Do you experience excessive tension and anxiety?	No	33	97.1	1	2.9	7.84	0.020
	Yes, during exams, only	122	93.1	9	6.9		
	Yes, since 1st year, I have had this problem	63	82.9	13	17.1		
Is your menstrual cycle regular?	No	33	78.6	9	21.4	8.40	0.004
	Yes	186	93.0	14	7.0		
If not, how long has it been delayed?	The last answer is yes	186	93.0	14	7.0	27.37	<0.001
	One month	26	89.7	3	10.3		
	Two months	6	66.7	3	33.3		
	Three or more months	1	25.0	3	75.0		
Do you have diabetes?	No	214	91.5	20	8.5	7.54	0.006
	Yes	5	62.5	3	37.5		
if yes, HbA1c	The previous answer is no	214	91.5	20	8.5	7.64	0.019
	Normal	2	66.7	1	33.3		
	Seven or above	3	60.0	2	40.0		
If yes – which type of diabetes?	The previous answer is no	214	91.5	20	8.5	7.64	0.022
	Type 1	3	60.0	2	40.0		
	Type 2	2	66.7	1	33.3		
	Gestational diabetes	0	0.0	0	0.0		
Do you have PCOS in your family	No	104	95.4	5	4.6	14.85	0.001
	Yes	20	71.4	8	28.6		
	I do not know	94	90.4	10	9.6		
BMI	Underweight	29	90.6	3	9.4	2.89	0.409
	Normal	136	91.9	12	8.1		
	Overweight	37	90.2	4	9.8		
	Obese	16	80.0	4	20.0		
How many times do you work out?	No, I did not	97	88.2	13	11.8	3.85	0.427
	Once	38	88.4	5	11.6		
	Twice	37	92.5	3	7.5		
	Three times	26	100.0	0	0.0		
	More than three times	21	91.3	2	8.7		
If the previous question is yes, what type of workout are you getting?	The previous answer is no	97	88.2	13	11.8	5.92	0.314
	Walk	114	92.7	9	7.3		
	Swimming	5	100.0	0	0.0		
	Basketball	1	50.0	1	50.0		
	Football	1	100.0	0	0.0		
	Cycling	1	100.0	0	0.0		
Have you heard about the relationship between lifestyle behavior and PCOS?	No	121	94.5	7	5.5	5.25	0.022
	Yes	97	85.8	16	14.2		
What kind of food do you eat?	Healthy	7	70.0	3	30.0	11.08	0.040
	Vary between healthy and unhealthy	189	93.1	14	6.9		
	Unhealthy	22	78.6	6	21.4		

## Discussion

PCOS is a common endocrine system disorder that affects reproductive-age women. We can also call it hyperandrogenic anovulation (HA); another name is Stein-Leventhal syndrome [15]. In our study, we aimed to provide insight into the prevalence of medical students with PCOS and to study the relationship between medical students' lifestyles and the increased prevalence of PCOS.

Our findings revealed that 23 (9.5%) out of 243 medical students at Taif University had been diagnosed with PCOS, and 156 (64.9%)of students reported suffering from acne and oily skin. Most symptoms increased during exams. In a study conducted at Dhaka Medical College Hospital (DMCH), researchers discovered that out of 40 women with acne, 11 (27.5%) were diagnosed with PCOS, eight (20%) exhibited PCOS features on ultrasound, and 10 women had an elevated LH to FSH ratio [16]. Furthermore, 36 students (14.9%) in our study suffer from blackish marks on the neck. In addition, medical female students experience generalized anxiety.

On the other hand, our study demonstrated that menstrual irregularity is one of the important risk factors for PCOS; however, most female students have regular menstrual cycles. According to the most recent recommendations, irregular menstrual periods can be used as a diagnostic marker of ovulatory dysfunction. Oligo-amenorrhea (cycles > 35 days apart or <8 cycles per year) is the most common type of menstrual dysfunction [17]. Considering obesity is one of the risk factors, most of the participants have normal BMI, but 41 (17%) participants were overweight and 20 (8.3%) were obese. For clarification, Basu et al. considered the various mechanisms that could be responsible for the relationship between increased adiposity and PCOS [18]. Increased adiposity played an important mechanism in the development of PCOS and significantly affected the severity of clinical and endocrine features in many women with the condition. The authors found that female patients with PCOS have higher α-amylase, which is secreted in stressful situations, than other people, and it is significantly associated with BMI [18]. Moreover, we investigated whether there is a relationship between medical students who are at risk or who have already been diagnosed with PCOS and their family history as a risk factor. We found that 28 (11.6%) answered they had a family history. Although there are no previous studies that support this finding, we still need to consider the possibility that a female medical student who has been diagnosed with PCOS or is at risk may have a family history of the syndrome.

In addition, out of the 23 students who were diagnosed with PCOS, eight (3.3%) have diabetes mellitus. In a previous study in Sahlgrenska University Hospital for infertility or hirsutism that compared the development of type 2 diabetes among females with PCOS and another control group without PCOS, researchers tracked a group of women with PCOS from around age 30 to over 50. The findings revealed women with PCOS were more likely to develop type 2 diabetes compared to those without PCOS (n=5 (19%) versus n=1 (1%) in the control group). Notably, all the women who developed type 2 diabetes were significantly overweight, and fat distribution was mainly around the waist [19]. These findings underscore the significance of early PCOS diagnosis. Additionally, women diagnosed with PCOS should receive guidance on weight management to mitigate the risk of developing type 2 diabetes. Therefore, healthcare professionals should closely track weight and waist measurements in all women with PCOS to assess their susceptibility to type 2 diabetes in the future.

Interestingly, our results showed a significant connection between the lifestyle behavior of medical students and PCOS. Our findings found that students who adhered to a healthy diet exhibited a higher incidence of PCOS compared to those with unhealthy or diverse eating patterns. However, students who exercised more than once a week showed a lower incidence of PCOS than those who did not participate in physical activity. Conversely, in a prior study that used an observational cross-sectional and case-control design, researchers investigated the relationship between physical activity and dietary patterns among adolescents with PCOS compared to healthy control girls. The study revealed notable differences in lifestyle between girls with PCOS and their age-matched control group. Specifically, Girls with PCOS consumed substantially more fat food than those in the control group, they also exhibited lower physical activity levels almost always. Interestingly, moderate to high physical activity significantly reduced the likelihood of PCOS [20].

These findings showed that the prevalence of PCOS is not significantly high, but most of students are at risk of developing PCOS due to the kind of lifestyle they had. Therefore, we recommended working to increase their awareness and improving good habits, such as exercising and eating healthy food is crucial. As well diabetic students should take care of hemoglobin (Hb)A1c, make a more nutritious food plan, regularly take their pills and insulin, and understand how to take the right dose. We suggested raising awareness among medical students using social media posters, university counseling advice programs, encouraging physical activity and dietary changes, and helping the campus community organize screenings.

Limitations

Despite the valuable insights and guidance, the study provided for gynecologists and other physicians, there are some limitations. The study cross-sectional nature exposed it to selection and recall biases. It was also single-centered, which limits the generalizability of the findings. Additionally, the treatment factor was not considered, constituting a potential variable.

## Conclusions

The study findings showed that Taif University medical students do not exhibit a noticeably increased prevalence of PCOS as most were of normal weight, non-diabetic, and had regular menstrual cycles. However, most students are at risk of developing PCOS due to unhealthy lifestyle behaviors, and anxiety, females with a positive family history of PCOS, females with irregularity in the menstrual cycle, and those who have diabetes. Thus, increasing awareness among medical students by promoting physical activity and changing eating habits, providing counseling programs at the university, using social media posters, and supporting the university community in running screenings are crucial. Further research and interventions are warranted to enhance the health and well-being of individuals affected by PCOS in Saudi Arabia.

## Appendices

**Table 4:** Questionnaire appendix 1

Questions	Options
Q1- Have you been diagnosed with PCOS?	•Yes •No
Q2- Do you suffer from acne and oily skin recently?	•Yes • No
Q3-If yes, what time is it increased?	•The previous answer no •During the exam period only •Along semester
Q4-Do you have a blackish mark around your neck?	•Yes •No
Q5-Do you experience excessive tension and anxiety?	•No •Yes, during the exam period only •Yes, since 1^st^ year I have had this problem
Q6-Is your menstrual cycle regular?	•Yes •No
Q7-If not, how long has it been delayed?	•The last answer is yes •Once month •Two months •Three or more months
Q8-Do you have diabetes?	•Yes •No
Q9-if yes, HbA1c	•The previous answer is no •Normal •Seven or above%
Q10-If yes – which type of diabetes?	•The previous answer is no •Type 1 •Type 2 •Gestational diabetes
Q11-Do you have PCOS in your family?	•Yes •No •I don’t know
(Q12-weight and height (BMI	- •Underweight (BMI<18.5 kg/m2) - •Normal (BMI 18.5–24.9 kg/m2) - •Overweight (BMI 25–29.9 kg/m2) - •Obese (BMI≥30 kg/m2)
Q13-How many times do you work out?	•No I did not • Once •Twice •Three times •More than three times
Q14-If the previous question is yes, what type of workout do you do?	•The previous answer is no •Walk •Swimming •Basketball •Football •Cycling
Q15-Have you heard about the relationship between lifestyle behavior and polycystic ovarian syndrome?	- •Yes •No
Q16-What kind of food do you eat?	•Healthy •Vary between healthy and •unhealthy •Unhealthy
